# Diagnostic Accuracy of Non-invasive Tests Versus Arthroscopy in Anterior Cruciate Ligament (ACL) Injuries

**DOI:** 10.7759/cureus.60925

**Published:** 2024-05-23

**Authors:** Junaid Zeb, Muhammad I Chaudary, Marwa Zeb, Mahmoud Mersal, Bilal Ahmad, Mohamed Alsonbaty

**Affiliations:** 1 Trauma and Orthopaedics, University Hospitals Birmingham, NHS Foundation Trust, Birmingham, GBR; 2 Trauma and Orthopaedics, Russells Hall Hospital, Dudley, GBR; 3 Radiology, Khyber Medical University (KMU), Peshawar, PAK; 4 Trauma and Orthopaedics, Queen Elizabeth Hospital Birmingham, Birmingham, GBR

**Keywords:** acl tear, acl, anterior drawer test, lachman test, mri, ligament rupture, anterior cruciate ligament

## Abstract

Background: The knee joint assessment to detect anterior cruciate ligament (ACL) injury after trauma involves clinical examination and radiography. The gold standard method is doing arthroscopy. We did this study seeking to evaluate the effectiveness of other non-invasive diagnostic methods, including the Anterior Drawer test, Lachman test and magnetic resonance imaging (MRI) in detecting ACL tears after trauma, compared to the reference standard method (the arthroscopy).

Methodology: This descriptive cross-sectional study was conducted in the Orthopaedic Department of the Khyber Teaching Hospital, Peshawar, for six months. A total of 86 participants with knee injuries fulfilling the inclusion criteria were recruited for the study. Mechanism of injury, side of injury and body weight were recorded. The Anterior Drawer test and Lachman test for ACL injury were performed by orthopaedic surgeons with at least five years of post-fellowship experience in orthopaedic surgery. Sensitivities, specificities and accuracy of the clinical tests and MRI were calculated.

Results: The statistical analysis revealed that the mean age of participants was 35.73 (SD 12.7) years, with a range from 18 to 55 years. Among the participants, 67 (77.91%) were male and 19 (22.09%) were female. The side of injury was predominantly right in 50 (58.14%) and left in 36 (41.86%) participants. Road traffic accidents (RTAs) were the leading cause of knee injury, accounting for 63.95% (55) of cases, followed by sports injuries at 23.26% (20).

Regarding diagnostic accuracy, MRI showed a sensitivity of 98.57%, specificity of 87.50% and diagnostic accuracy of 96.51% in detecting ACL tears. The Lachman test demonstrated a sensitivity of 90%, specificity of 87.5% and diagnostic accuracy of 89.53% compared to arthroscopy. Similarly, the anterior Drawer test exhibited a sensitivity of 88.57%, specificity of 87.50% and diagnostic accuracy of 88.37% against the gold standard of arthroscopy. These findings underscore the effectiveness of these diagnostic modalities in identifying ACL injuries.

Conclusions: All three tests (MRI, Lachman test and anterior Drawer test) can be used for the diagnosis of anterior cruciate ligament injury with optimal results.

## Introduction

The knee joint, one of the most frequently involved joints in trauma, consists of two cruciate ligaments and two menisci [1]. Assessment of the knee joint is done conventionally by clinical examination and radiography [2]. In the modern era, magnetic resonance imaging (MRI) and arthroscopy are investigations of choice for knee trauma [3], complemented by history and clinical examination, including special tests for ligaments and menisci [4]. These special clinical tests are of immense importance in diagnosing knee injuries with sensitivity ranging from 35% to 70% [1].

The importance of MRI and clinical examination is its non-invasiveness. On the other hand, arthroscopy is a keyhole surgery that can even be performed as a daycare procedure, depending on its indication. Arthroscopy is the gold standard procedure, and its accuracy reaches up to 100%. But because of its invasiveness, it's not without complications. On the other hand, MRI and clinical examination are of key importance as being non-invasive [5-7].

Literature showed that the sensitivity and specificity of the anterior draw test were found as 94.4% and 96.4% with and without anaesthesia, respectively. While sensitivity for the Lachman test with and without anaesthesia was 93.5% and 96.9%, respectively [8].

Another study reported the Lachman test as 81% sensitive and 87% specific for detecting anterior cruciate ligament (ACL) injuries without anaesthesia, while with anaesthesia its sensitivity and specificity were increased to 91% and 78%, respectively, which showed a significant fall in specificity with anaesthesia. The anterior Drawer test sensitivity and specificity were only 38% and 81%, respectively, without anaesthesia, which increased to 63% and 91% with anaesthesia [9]. Others reported sensitivity and specificity of anterior drawer and Lachman tests without anaesthesia as 89.3% and 100%, and 78.6% and 100%, respectively, which was significantly increased to 92.9% and 100% for each abovementioned tests under anaesthesia [10].

Clinical examination with special tests is 25% to 70% effective in diagnosing pathology in knee injuries. However, in acute settings, clinical tests may not be appropriate due to pain. Hence, MRI is preferred as it is non-invasive and highly sensitive for meniscal injuries but unfortunately less sensitive for ACL injuries [11]. Its sensitivity was reported as 87.87% in ACL tears [12]. MRI is completely non-invasive and provides the best opportunity to visualise all articular ligaments, even extra-articular ligaments where even arthroscopy fails to visualise them [13].

The technique of arthroscopy is much more traumatic and requires more expenses and skills. In case of favourable results, we can replace arthroscopy with a non-invasive, less expensive and easily available technique of MRI. Although international literature has documented the diagnostic value of MRI in diagnosing knee joint ligamentous injuries. However, the results can be different from other populations in terms of genetic and environmental factors. Moreover, local studies regarding MRI accuracy have taken a very small sample size [14,15], so more data need to be available to establish the proper accuracy of MRI in ACL injuries. If the accuracy of MRI is found to be 80% or more with a high positive predictive value, we can decrease the burden of arthroscopies as it is an invasive procedure performed under anaesthesia [14].

This study was conducted to determine the accuracy of non-invasive tests, i.e., the anterior Drawer test, Lachman test and MRI study in the diagnosis of ACL tear in comparison to arthroscopy taken as the gold standard.

## Materials and methods

This descriptive cross-sectional study was conducted in the Inpatient Department of Orthopaedics at Khyber Teaching Hospital, Peshawar, from October 1, 2021, to March 1, 2022. The Institutional Review Board (IRB) of Khyber Medical College (KMC), Peshawar, approved the study with approval number 983/DME/KMC.

The sample size was 86. For the anterior Drawer test, the expected sensitivity was 94.4% and the expected specificity was 96.4%. Keeping the β-error (precision) at 5% and the α-error (confidence interval) at 95%, the sample size can be calculated using the following formulas [16]:

 *N* = (*Z*^2 ^x [(*S_n_* (1 - *S_n_*)])/*E*^2^= (1.96)^2^ x (0.94 x 0.06)/(0.05)^2^ = 86

Patients with knee injuries of either gender whose age was from 18 to 55 years and suspected ACL injury upon clinical examination or MRI or history, who were admitted to the ward for arthroscopic examination, were included in the study. Patients with associated fractures of the femoral condyle, tibial plateau, dislocation of the knee joint on plain radiograph, who underwent previous knee surgeries or osteoarthritic knees and those having any contraindications for MRI imaging were excluded from the study.

Data were collected by non-probability consecutive sampling technique after seeking permission from the hospital's ethical committee. A detailed history was taken from patients, and then a physical examination was performed. The anterior Drawer test and Lachman test were performed for ACL injury. These clinical tests were performed by orthopaedic surgeons who had at least five years of post-fellowship experience in orthopaedic surgery. Data analysis was performed using IBM SPSS Statistics for Windows, Version 22.0 (IBM Corp., Armonk, NY). For continuous variables like age and body weight mean and standard deviation were calculated, while frequency and percentages were calculated for gender, affected side (right, left or both knees), mechanism of injury and findings on MRI and arthroscopy. Initially, false positive, false negative, true positive and true negative were found via crosstabs using IBM SPSS Statistics for Windows, Version 22.0. These values were entered into MEDCALC online software (https://www.medcalc.org/calc/diagnostic_test.php) for calculating sensitivities, specificities and accuracy of the clinical tests and MRI. The level of significance was *P *≤ 0.05.

## Results

The mean age of participants was 35.73 (SD 12.7) years, with a range from 18 to 55 years. There were 67 males (77.91%) and 19 females (22.09%). The side of injury was right in 50 (58.14%) and left in 36 (41.86%) participants. The most common cause of knee injury was RTA in 55 (63.95%) followed by sports injury in 20 (23.26%) participants (Table 1).

**Table 1:** Baseline characteristics. RTA, road traffic accident

Variable	Characteristics	Frequency (%)
Gender	Female	19 (22.09)
	Male	67 (77.91)
Side of injury	Left	36 (41.86)
	Right	50 (58.14)
Mechanism of injury	Fall	8 (9.30)
	Occupational injury	3 (3.49)
	RTA	55 (63.95)
	Sport injury	20 (23.26)

The number of participants was highest in the age group 18-30 years (*n *= 34, 39.53%), followed by the age group 31-40 (*n *= 23, 26.74%). The least number of participants were in the age group 41-50 years (*n *= 13, 15.12%).

The sensitivity, specificity and diagnostic accuracy of MRI in detecting ACL tears were 98.57%, 87.50% and 96.51%, respectively. The positive likelihood ratio, negative likelihood ratio, positive predictive value and negative predictive value with 95% confidence intervals (CIs) of MRI are given in Table 2.

**Table 2:** Diagnostic statistics of magnetic resonance imaging (MRI), Lachman test and anterior Drawer test versus arthroscopy. Data analysis was performed using IBM SPSS Statistics for Windows, Version 22.0.

Statistics	MRI	Lachman test	Anterior Drawer test
Sensitivity (%)	98.57	90.00	88.57
Specificity (%)	87.50	87.50	87.50
Positive likelihood ratio	7.89	7.2	7.09
Negative likelihood ratio	0.02	0.11	0.13
Positive predictive value (%)	97.18	81.40	81.40
Negative predictive value (%)	93.33	96.92	96.87
Diagnostic accuracy (%)	96.51	89.53	88.37

The diagnostic accuracy of the Lachman test versus gold standard arthroscopy shows that sensitivity was 90%, specificity was 87.5% and diagnostic accuracy was 89.53%. Moreover, the sensitivity, specificity and diagnostic accuracy of the anterior Drawer test against gold standard arthroscopy were 88.57%, 87.50% and 88.37%, respectively (Table 2).

**Figure 1:**
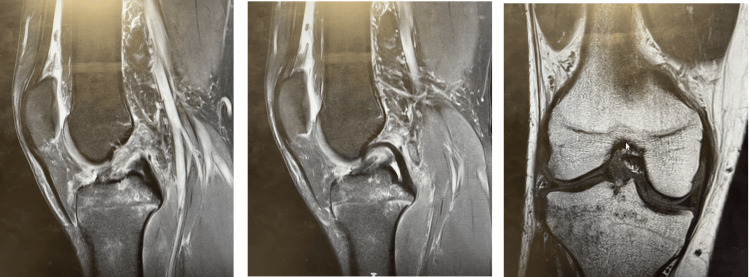
Sagittal and coronal magnetic resonance imaging (MRI) scans of a 34-year-old male patient showing torn anterior cruciate ligament (ACL).

MRI scans for a 34-year-old male with an ACL tear are shown in Figure 1.

**Figure 2:**
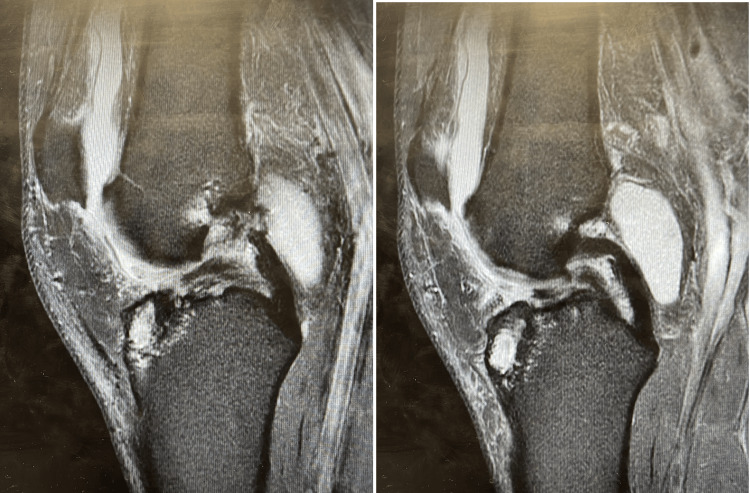
T2-weighted sagittal magnetic resonance imaging (MRI) scans of a 46-year-old male patient showing torn anterior cruciate ligament (ACL).

MRI scans for a 34-year-old male with an ACL tear are shown in Figure 2.

## Discussion

Our study showed that MRI, anterior Drawer test and Lachman test have high diagnostic accuracy in the diagnosis of ACL injury. The ACL injury diagnosis might be laid out in various ways: clinical assessment, clinical imaging or arthroscopic evaluation. Arthroscopic evaluation is most widely acknowledged as the gold standard for determining ACL injury; however, it is an invasive procedure with associated surgical risks. The precision of finding through arthroscopy is reliant upon the ability of the clinician [17,18]. In our study, we used arthroscopy as the gold standard for diagnosing ACL tear. According to currently available data, its sensitivity and specificity for assessing knee injury reach up to 100%. However, as it is invasive, it is not without complications, such as infection, hemarthrosis, future adhesions and sympathetic dystrophy, especially in the long term [6,7].

Our findings showed that the sensitivity, specificity and diagnostic accuracy of MRI in detecting ACL tears were 98.57%, 87.50% and 96.51%, respectively. MRI is ideal for imaging the complex anatomy of the knee joint [4,5]. Its sensitivity was reported as 87.87% in ACL tears [12], which was comparable to our study. Another systematic review contrasting arthroscopy with MRI containing 2,040 participants having ACL injury reported 93.4% accuracy, 86.5% sensitivity, 95.2% specificity, 82.9% positive predictive value and 96.4% negative predictive value for MRI [19]. Similarly, a meta-analysis by Oei et al. reported a high specificity (94.3%) and sensitivity (94.4%) for MRI in the diagnosis of ACL. This meta-analysis analysis of the funnel plot revealed publication bias probably not going to be a critical component in making the outcomes stronger [20]. Another study on 78 participants compared the diagnostic accuracy of MRI against the gold standard of arthroscopy and reported that the sensitivity, specificity and accuracy of MRI in the diagnosis of ACL injury were 95.45%, 91.67% and 94.87%, respectively [21].

Our findings showed that the diagnostic accuracy of the Lachman test versus gold standard arthroscopy showed that sensitivity was 90%, specificity was 87.5% and diagnostic accuracy was 89.53%. A study of 45 participants on the diagnostic accuracy of the Lachman test versus gold standard arthroscopy showed that sensitivity was 81%, specificity was 87.5% and diagnostic accuracy was 93% [22]. These results were consistent with our study. Another study evaluated the sensitivity and specificity of the Lachman test showing sensitivity and specificity of 89.3% and 100%, respectively [10].

Meta-analyses and systematic reviews have reliably found the Lachman test to have high sensitivity and accuracy for the diagnosis of an ACL tear [9]. A meta-analysis by Benjaminse et al. on 28 papers recognised issues with heterogeneity, with all studies having at least a chance of bias due to weak study designs. However, it found high diagnostic accuracy for the Lachman test [23]. van Eck et al. [9] conducted a meta-analysis focusing on short-duration (<3 weeks) wounds of ACL. They revealed that the Lachman test had 81% sensitivity when performed without sedation [24]. However, others showed this test was not superior in isolation [25]. Also, there is insufficient evidence that an unskilled person performed this test [26]. The best practice guidelines showed that the Lachman test was superior to the anterior Drawer test in the diagnosis of ACL injury [25].

Our results showed that the sensitivity, specificity and diagnostic accuracy of the anterior Drawer test against gold standard arthroscopy were 88.57%, 87.50% and 88.37%, respectively. A previous study showed that the anterior Drawer test sensitivity and specificity were only 38% and 81%, respectively, without anaesthesia, while under anaesthesia, sensitivity and specificity were improved to 63% and 91%, respectively [9]. A meta-analysis on the comparison of clinical tests against the gold standard arthroscopy in the diagnosis of ACL tear included 22 studies that reported that the sensitivity and specificity, respectively, for the anterior Drawer test were 83% and 85%, respectively [27]. Our results were inconsistent with previous literature as explained.

Study limitations

The study has some limitations, such as being conducted at a single centre and having a relatively small sample size. More studies in multiple centres with a larger number of participants are recommended to further explore this area.

## Conclusions

Within the limitation of our study, as it was done within one centre and with a relatively small patient sample, it can be concluded that our study emphasises the practical and reliable role of all three non-invasive methods (the MRI, the Lachman test and the anterior Drawer test) in diagnosing ACL injuries. All of them can be used for the diagnosis of anterior cruciate ligament injury with optimal results.

Future research efforts aiming for larger-scale studies involving multiple centres and large patient samples are recommended. By expanding the scope of the investigation, we can further validate the effectiveness and accuracy of these non-invasive tests in the diagnosis of ACL injuries.

## References

[1] Kostov H, Kaftandziev I, Arsovski O, Kostova E, Gavrilovski A (2014). Clinical outcomes of three different modes of femoral hamstring graft fixation in anterior cruciate ligament reconstruction. Macedonian Med Rev.

[2] Bronstein RD, Schaffer JC (2017). Physical examination of knee ligament injuries. J Am Acad Orthop Surg.

[3] Koster CH, Harmsen AM, Lichtenberg MC, Bloemers FW (2018). ACL injury: How do the physical examination tests compare?. J Fam Pract.

[4] Refaat M, El Shazly E, Elsayed A (2020). Role of MR imaging in evaluation of traumatic knee lesions. Benha Med J.

[5] Khandelwal K, Chaturvedi V, Mishra V, Khandelwal G (2018). Diagnostic accuracy of MRI knee in reference to arthroscopy in meniscal and anterior cruciate ligament injuries. Egypt J Radiol Nucl.

[6] Vijay C, Supreeth N, Ravishankar R, Vardhan RV, Vanaja GS (2018). Clinical, magnetic resonance imaging, and arthroscopic correlation in anterior cruciate ligament and meniscal injuries of the knee. J Orthop Trauma Rehab.

[7] Panigrahi R, Priyadarshi A, Palo N, Marandi H, Agrawalla DK, Biswal MR (2016). Correlation of clinical examination, MRI and arthroscopy findings in menisco-cruciate injuries of the knee: a prospective diagnostic study. Arch Trauma Res.

[8] Makhmalbaf H, Moradi A, Ganji S, Omidi-Kashani F (2013). Accuracy of lachman and anterior drawer tests for anterior cruciate ligament injuries. Arch Bone Jt Surg.

[9] van Eck CF, van den Bekerom MP, Fu FH, Poolman RW, Kerkhoffs GM (2013). Methods to diagnose acute anterior cruciate ligament rupture: a meta-analysis of physical examinations with and without anaesthesia. Knee Surg Sports Traumatol Arthrosc.

[10] Jain DK, Amaravati R, Sharma G (2009). Evaluation of the clinical signs of anterior cruciate ligament and meniscal injuries. Indian J Orthop.

[11] Mohammed E. R., Metwally N. A. E. S., & Elaidy A. A. S (2019). Role of magnetic resonance imaging in assessment of anterior cruciate ligament post-grafting cases in terms of graft integrity and complications. Egyptian J Hosp Med.

[12] Bari AA, Kashikar SV, Lakhkar BN, Ahsan MS (2014). Evaluation of MRI versus arthroscopy in anterior cruciate ligament and meniscal injuries. J Clin Diagn Res.

[13] Li K, Du J, Huang LX, Ni L, Liu T, Yang HL (2017). The diagnostic accuracy of magnetic resonance imaging for anterior cruciate ligament injury in comparison to arthroscopy: a meta-analysis. Sci Rep.

[14] Rahman A, Nafees M, Akram MH, Andrabi AH, Zahid M (2010). Diagnostic accuracy of magnetic resonance imaging in meniscal injuries of knee joint and its role in selection of patients for arthroscopy. J Ayub Med College.

[15] Khanda GE, Akhtar W, Ahsan H, Ahmad N (2008). Assessment of menisci and ligamentous injuries of the knee on magnetic resonance imaging: correlation with arthroscopy. J Pak Med Assoc.

[16] Domnick C, Raschke MJ, Herbort M (2016). Biomechanics of the anterior cruciate ligament: physiology, rupture and reconstruction techniques. World J Orthop.

[17] Salzler MJ, Lin A, Miller CD, Herold S, Irrgang JJ, Harner CD (2014). Complications after arthroscopic knee surgery. Am J Sports Med.

[18] Sapsford H, Sutherland AG (2016). Reducing time to surgery after anterior cruciate ligament injury. Scott Med J.

[19] Leblanc MC, Kowalczuk M, Andruszkiewicz N (2015). Diagnostic accuracy of physical examination for anterior knee instability: a systematic review. Knee Surg Sports Traumatol Arthrosc.

[20] Oei EH, Nikken JJ, Verstijnen AC, Ginai AZ, Myriam Hunink MG (2003). MR imaging of the menisci and cruciate ligaments: a systematic review. Radiology.

[21] Zhao M, Zhou Y, Chang J (2020). The accuracy of MRI in the diagnosis of anterior cruciate ligament injury. Ann Transl Med.

[22] Mulligan EP, McGuffie DQ, Coyner K, Khazzam M (2015). The reliability and diagnostic accuracy of assessing the translation endpoint during the Lachman test. Int J Sports Phys Ther.

[23] Benjaminse A, Gokeler A, van der Schans CP (2006). Clinical diagnosis of an anterior cruciate ligament rupture: a meta-analysis. J Orthop Sports Phys Ther.

[24] Meuffels DE, Poldervaart MT, Diercks RL (2012). Guideline on anterior cruciate ligament injury. Acta Orthop.

[25] CKS is only available in the UK. (2017 (2017). CKS is only available in the UK. https://www.nice.org.uk/cks-uk-only#!scenario:1.

[26] Thorlund JB, Juhl CB, Roos EM, Lohmander LS (2015). Arthroscopic surgery for degenerative knee: systematic review and meta-analysis of benefits and harms. BMJ.

[27] Sokal PA, Norris R, Maddox TW, Oldershaw RA (2022). The diagnostic accuracy of clinical tests for anterior cruciate ligament tears are comparable but the Lachman test has been previously overestimated: a systematic review and meta-analysis. Knee Surg Sports Traumatol Arthrosc.

